# Glycemic variability and the risk of atrial fibrillation: a meta-analysis

**DOI:** 10.3389/fendo.2023.1126581

**Published:** 2023-05-18

**Authors:** Wei Li, Yang Wang, Guoqiang Zhong

**Affiliations:** Department of Cardiology, The First Affiliated Hospital of Guangxi Medical University, Nanning, China

**Keywords:** glycemic variability, atrial fibrillation, risk factor, meta-analysis, incidence

## Abstract

**Background:**

Glycemic variability (GV) has been associated with vascular complications in patients with diabetes. However, the relationship between GV and risk of atrial fibrillation (AF) remains not fully determined. We therefore conducted a systematic review and meta-analysis to evaluate the above association.

**Methods:**

Medline, Embase, Web of Science, Wanfang, and China National Knowledge Infrastructure were searched for longitudinal follow-up studies comparing the incidence of AF between patients with higher versus lower GV. A random-effects model incorporating the potential heterogeneity was used to pool the results.

**Results:**

Nine cohort studies with 6,877,661 participants were included, and 36,784 (0.53%) participants developed AF during follow-up. Pooled results showed that a high GV was associated with an increased risk of AF (risk ratio [RR]: 1.20, 95% confidence interval [CI]: 1.11 to 1.30, p < 0.001, I^2^ = 20%). Subgroup analyses suggested consistent association between GV and AF in prospective (RR: 1.29, 95% CI: 1.05 to 1.59, p = 0.01) and retrospective studies (RR: 1.18, 95% CI: 1.08 to 1.29, p = 0.002), in diabetic (RR: 1.24, 95% CI: 1.03 to 1.50, p = 0.03) and non-diabetic subjects (RR: 1.13, 95% CI: 1.00 to 1.28, p = 0.05), in studies with short-term (RR: 1.25, 95% CI: 1.11 to 1.40, p < 0.001) and long-term GV (RR: 1.18, 95% CI: 1.05 to 1.34, p = 0.006), and in studies with different quality scores (p for subgroup difference all > 0.05).

**Conclusion:**

A high GV may predict an increased risk of AF in adult population.

## Introduction

Atrial fibrillation (AF) is one of the most common arrhythmia, particularly in older population (1, 2). Although in recent decades, continuous efforts have been made to develop novel treatments for AF, substantial patients with AF still have poor clinical outcomes, such as a higher incidence of stroke and increased overall mortality (3, 4). Therefore, identification of risk factors for AF is important for the prevention of AF and related adverse events (5).

Accumulating evidence from clinical observations suggests that diabetes may be a risk factor of AF (6, 7). In diabetes patients, persistent hyperglycemia evidenced by elevated glycosylated hemoglobin (HbA1c) is widely accepted as being responsible for cardiovascular complications (8). Subsequent investigations suggest that besides persistent hyperglycemia, hypoglycemia may also be independently associated with the risk of cardiovascular events in patients with diabetes, including AF (9, 10). The concept of glycemic variability (GV) is therefore raised during the recent decades, which reflects the extent of glucose fluctuation within days (short-term GV) and months/years (long-term GV) (11). Independent of persistent hyperglycemia, high GV has been shown to be a predictor of adverse cardiovascular events in both the diabetic and the non-diabetic population (12). Indeed, a high GV has been related to a higher incidence of coronary artery disease (CAD) (13), stroke (14), and overall cardiovascular mortality (15). Besides, an increased acute GV has also recently been identified as a potential predictor of poor survival in patients with sepsis (16). However, previous studies investigating the relationship between GV and AF showed inconsistent results (17). Some studies showed that a high GV may be a risk factor of AF (18–23), while other studies did not support this association (24–26). Therefore, in this study, we conducted a systematic review and meta-analysis to systematically evaluate the association between GV and the incidence of AF.

## Methods

The meta-analysis was performed in accordance with the MOOSE (Meta-analysis of Observational Studies in Epidemiology) (27) and Cochrane’s Handbook (28) guidelines.

### Literature search

Studies were identified *via* systematic search of electronic databases including Medline, Embase, Web of Science, Wanfang, and China National Knowledge Infrastructure (CNKI) from inception to September 19, 2022. A combined search strategy was used, which included: (1) “glycemic” OR “glyceamic” OR “glucose” OR “hemoglobin A1c” OR “A1C”‘; (2) “variability” OR “variation” OR “fluctuation”; and (3) “atrial fibrillation” OR “AF”. The search was limited to clinical studies published in English. The reference lists of related original and review articles were also analyzed using a manual approach.

### Study selection

The inclusion criteria for the studies were: (1) observational studies that are designed to follow up over time, such as cohort studies, *post-hoc* analyses of clinical trials, or nested case-control studies; (2) included adult population who were evaluated for GV at baseline; (3) incidence of AF during follow-up was observed and recorded; and (4) the incidence of AF between patients with higher versus lower GV was compared and reported as risk ratio (RR) and corresponding 95% confidence interval [CI], or these data could be calculated or estimated. Measuring methods and definition of GV were consistent with the criteria applied among the included studies. Reviews, editorials, preclinical studies, cross-sectional studies, studies that did not evaluate GV, studies that did not report the incidence of AF, or other studies that were irrelevant to the aim of the meta-analysis were excluded.

### Data extracting and quality evaluation

Literature search, data extraction, and quality assessment of the included studies were independently performed by two authors according to the predefined criteria. Discrepancies were resolved by consensus or discussion with the corresponding author. The extracted data included: (1) general information of the study (author, year, and country); (2) study design characteristics; (3) patient characteristics (diagnosis, age, sex, and diabetic status); (4) exposure characteristics (parameter for GV, cutoff for defining high GV, and number of subjects with high GV at baseline); (5) follow-up and outcome (follow-up durations, methods for validation the diagnosis of AF, and number of patients with AF incidence); and (6) potential confounding factors that were adjusted or matched when the association between GV and the incidence of AF was estimated. The quality of each study was evaluated using the Newcastle-Ottawa Scale (29) which ranges from 1 to 9 stars and judges each study regarding three aspects: selection of the study groups; the comparability of the groups; and the ascertainment of the outcome of interest.

### Statistical analyses

The methods of the statistics are generally consistent with those used in the previous meta-analysis of cohort studies (30). We used RR and corresponding 95% CI as the general measure for association between GV at baseline and the incidence of AF during follow-up. Data of RR and the corresponding stand error (SE) were calculated from 95% CI or P values, and were logarithmically transformed to stabilize the variance and normalize the distribution (28). The Cochrane’s Q test was used to evaluate the heterogeneity among the included studies (31). Besides, the I^2^ statistic was also estimated (31). A significant heterogeneity was considered if I^2^ > 50%. We used a random-effect model to synthesize the RR data because this model is considered as a more generalized method which incorporates the potential heterogeneity among the included studies (32). Sensitivity analysis was used to evaluate the possible influence of each study on the pooled results (33). Predefined subgroup analyses were also performed to evaluate the influences of study characteristics on the association, such as study design, diabetic status of the participants, type of GV (short-term or long-term), and quality scores of the studies. The potential publication bias was assessed by funnel plots with the Egger’s regression asymmetry test (34). A P value < 0.05 indicates statistically significance. We used the RevMan (Version 5.1; Cochrane Collaboration, Oxford, UK) and Stata 12.0 software for the meta-analysis and statistical analysis.

## Results

### Literature search

**Figure 1:**
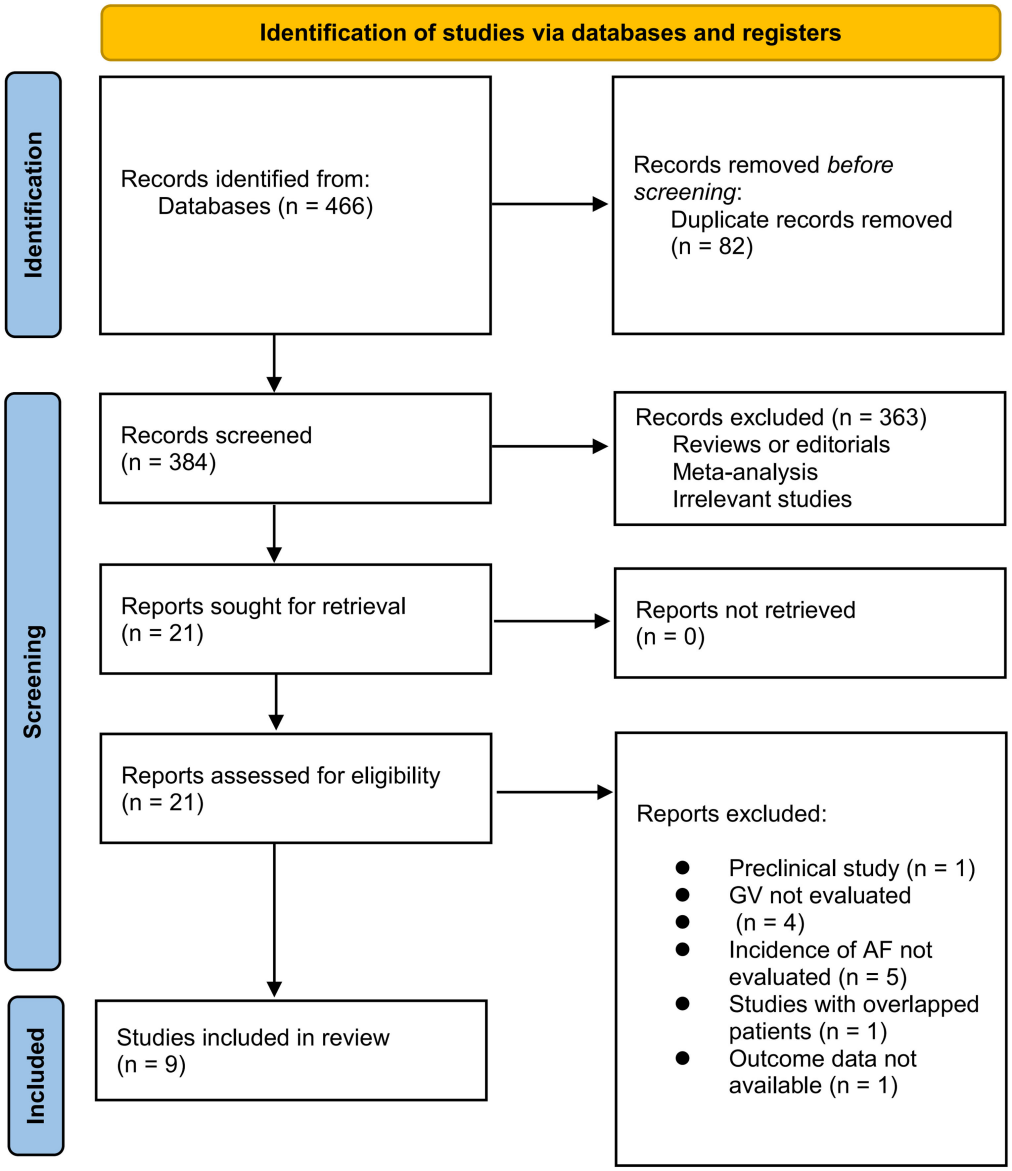
Flowchart of literature search and study retrieval.

The process of database search is summarized in **Figure 1**. Briefly, 466 articles were found *via* initial literature search, and 384 articles were left after excluding the duplicated records. Subsequently, 363 articles were excluded by screening the titles and abstracts because they were not relevant to the objective of the meta-analysis. Accordingly, 21 articles underwent full-text review, and 12 of them were further excluded for the reasons listed in **Figure 1**. Finally, 9 studies were included in the meta-analysis (18–26).

### Study characteristics and quality evaluation

The characteristics of the included studies are summarized in **Table 1**. Overall, nine studies, including 3 prospective cohort studies (19, 25, 26) and 6 retrospective cohort studies (18, 20–24), were included in the meta-analysis. These studies were published between 2012 and 2021, and carried out in China (18, 19, 22–24, 26), Singapore (25), Korea (21), and the United States (20). Fours studies included patients with CAD who underwent coronary artery bypass graft (CABG) (20, 24–26), one study included patients with acute coronary syndrome (ACS) (19), three studies included subjects with diabetes (18, 22, 23), and the remaining one study included healthy population (21). The mean ages of the patients varied between 59.3 and 68.2 years, and the proportions of men ranged from 16.4% to 80.2%. Various GV parameters were applied in the included studies, such as the mean or largest amplitude of glycemic excursion (MAGE or LAGE) for as the indicators of short-term GV (19, 20, 24–26), and the standard deviation (SD) or coefficient of variation (CV) of HbA1c or blood glucose (BG) as the indicators of long-term GV (18, 21–23). The follow-up durations were within hospitalization or 30 days for studies with short-term GV, and from 5.3 to 9.1 years for studies with long-term GV. Patients with incidental AF were generally validated with electrocardiograph or Holter, and a total of 36,784 (0.53%) patients developed AF during follow-up. Possible confounding factors, such as age, sex, smoking, alcohol drinking, and comorbidities were adjusted to a varying degree among the included studies. The NOS scores of the included studies ranged from six to eight, indicating moderate to good study quality (**Table 2**).

**Table 1:** Characteristics of the included cohort studies.

Study	Country	Design	Participants	No. of patients	Mean age (years)	Men (%)	DM (%)	Definition of high GV	Duration for GV measurements	Number of participants with high GV	Follow-up duration	AF validation	Number of AF cases	Variables adjusted or matched
**Su 2012** (24)	China	RC	Patients undergoing CABG	116	NR	NR	0	LAGE > 2.2 mmol/L	During hospitalization	40	30 days	ECG or Holter evidenced AF	15	Age, sex, and surgery type
**Gu 2017** (18)	China	RC	Patients with T2DM	505	68.2	59.8	100	HbA1c-SD > 0.66% or HbA1c-CV > 9.12%	At least 3 HbA1c values during follow-up	NR	6.9 years	ECG or Holter evidenced AF	48	Age, sex, LAD, LVMI, and BMI
**Xia 2017** (19)	China	PC	Patients with ACS	864	67	63.3	53.1	SDBG > 2 mmol/L	During hospitalization	275	30 days	ECG or Holter evidenced AF	94	Age, sex, comorbidities, smoking, and concurrent medications
**Sim 2018** (25)	Singapore	PC	Patients undergoing CABG	1743	59.3	80.2	46.6	LAGE > 6 mmol/L	Within 48 h after surgery	657	30 days	ECG or Holter evidenced AF lasting for > 1 hour	256	Age, sex, ethnicity, and surgery type
**Clement 2019** (20)	USA	RC	Patients undergoing CABG	2073	64.3	16.4	47.6	MAGE (median)	Within 24h after surgery	1036	During hospitalization	ECG or Holter evidenced AF lasting for > 1 hour	446	Age, sex, STS, HbA1c, surgery type, and blood transfusion
**Lee 2020** (21)	Korea	RC	Healthy population	6819829	NR	NR	0	SDBG (Q4)	During follow-up	1709474	5.3 years	ECG or Holter evidenced AF	31302	Age, sex, smoking, alcohol consumption, regular exercise, BMI, SBP, TC level, FBG, and income
**Lee 2021** (23)	China	RC	Patients with diabetes	25186	63	50.4	100	SDBG (Q4)	During follow-up	NR	9.1 years	ECG or Holter evidenced AF lasting for > 1 hour	1846	Age, sex, HbA1c, TC, HDL-c, and antidiabetic treatments
**Hsu 2021** (22)	China	RC	Patients with T2DM	27246	66.7	52.8	100	CVBG (Q4)	During follow-up	6812	5.9 years	Diagnostic code or ECG evidenced AF	2762	Age, sex, baseline BMI, HTN, COPD, CAD, PAOD, history of TIA/ischemic stroke, baseline FBG, baseline HbA1c, baseline eGFR, LA size, LVEF, LV mass, and medications
**Yan 2021** (26)	China	PC	Patients undergoing CABG	99	62.1	69.2	28.1	LAGE > 6 mmol/L	Within 48 h after surgery	36	During hospitalization	ECG or Holter evidenced AF lasting for > 1 hour	15	Age, sex, and minimal HGB during hospitalization

GV, glycemic variability; AF, atrial fibrillation; RC, retrospective cohort; PC, prospective cohort; CABG, coronary artery bypass graft; ACS, acute coronary syndrome; T2DM, type 2 diabetes mellitus; LAGE, largest amplitude of glycemic excursion; SDBG, standard deviation of blood glucose; CVBG, coefficient of variation of blood glucose; HbA1c, glycosylated hemoglobin; HbA1c-SD, standard deviation of HbA1c; HbA1c-CV, coefficient of variation of HbA1c; MAGE, mean amplitude of glycemic excursion; LAD, left atrial dimension; LVMI, left ventricular massive index; BMI, body mass index; STS, society of thoracic surgery score; SBP, systolic blood pressure; TC, total cholesterol; FBG, fasting blood glucose; HDL-c, high-density lipoprotein cholesterol; HGB, hemoglobin; HTN, hypertension; COPD, coronary obstructive pulmonary disease; CAD, coronary artery disease; PAOD, peripheral arterial occlusive disease; TIA, transient ischemic attack; eGFR, estimated glomerular filtrating rate; LA, left atrial; LV, left ventricular; LVEF, left ventricular ejection fraction. NR, not reported.

**Table 2:** Quality of the included studies evaluated *via* the Newcastle-Ottawa Score.

Study	Representativeness of the exposed cohort	Selection of the non-exposed cohort	Ascertainment of exposure	Outcome not present at baseline	Control for age and sex	Control for other confounding factors	Assessment of outcome	Enough long follow-up duration	Adequacy of follow-up of cohorts	Total
**Su 2012** (24)	0	1	1	1	1	1	1	0	1	7
**Gu 2017** (18)	0	1	1	1	1	1	1	1	1	8
**Xia 2017** (19)	1	1	1	1	1	1	1	0	1	8
**Sim 2018** (25)	1	1	1	1	1	1	1	0	1	8
**Clement 2019** (20)	0	1	1	1	1	1	1	0	1	7
**Lee 2020** (21)	0	1	1	1	1	1	1	1	1	8
**Lee 2021** (23)	0	1	1	1	1	1	1	1	1	8
**Hsu 2021** (22)	0	1	1	1	1	1	1	1	1	8
**Yan 2021** (26)	0	1	1	1	1	0	1	0	1	6

### GV and the risk of AF

**Figure 2:**
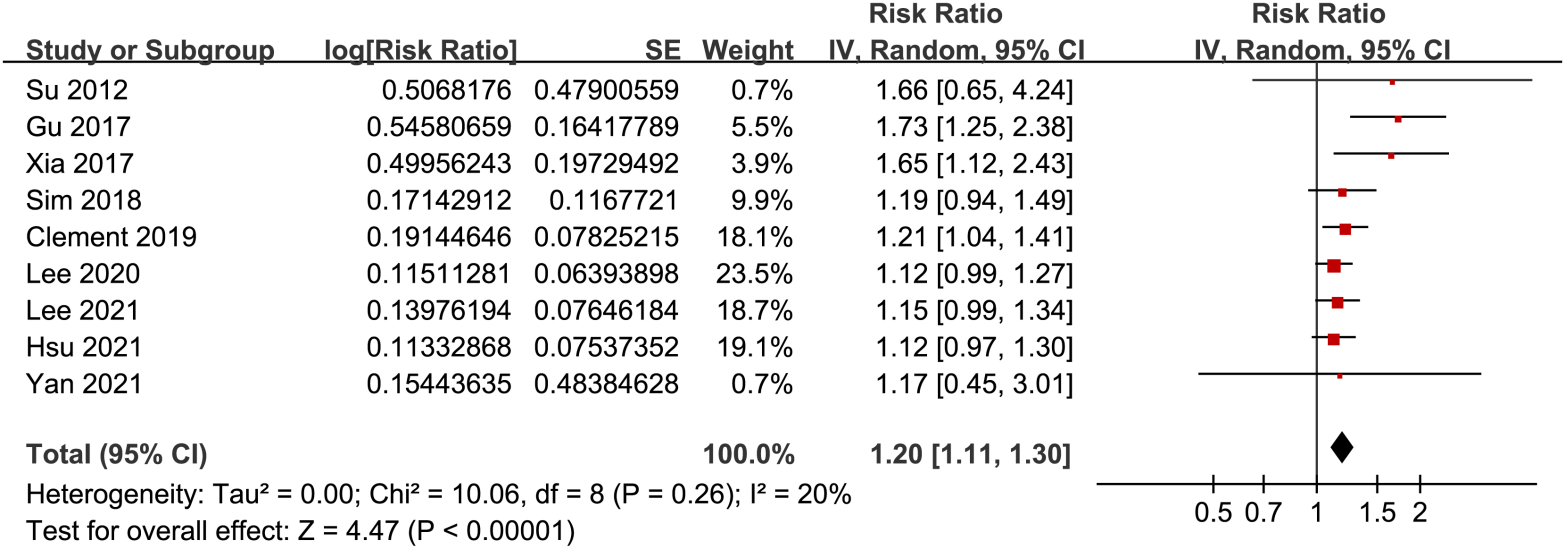
Forest plots for the meta-analysis of the association between GV and the risk of AF.

**Figure 3:**
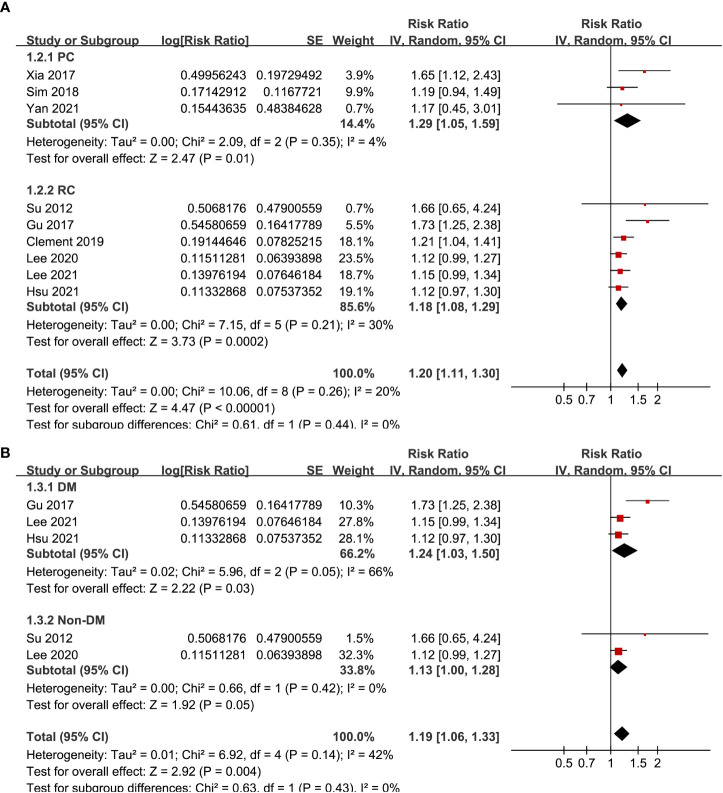
Subgroup analyses for the meta-analysis of the association between GV and the risk of AF. **(A)** subgroup analysis according to the study design; and **(B)** subgroup analysis according to the diabetic status of the subjects.

**Figure 4:**
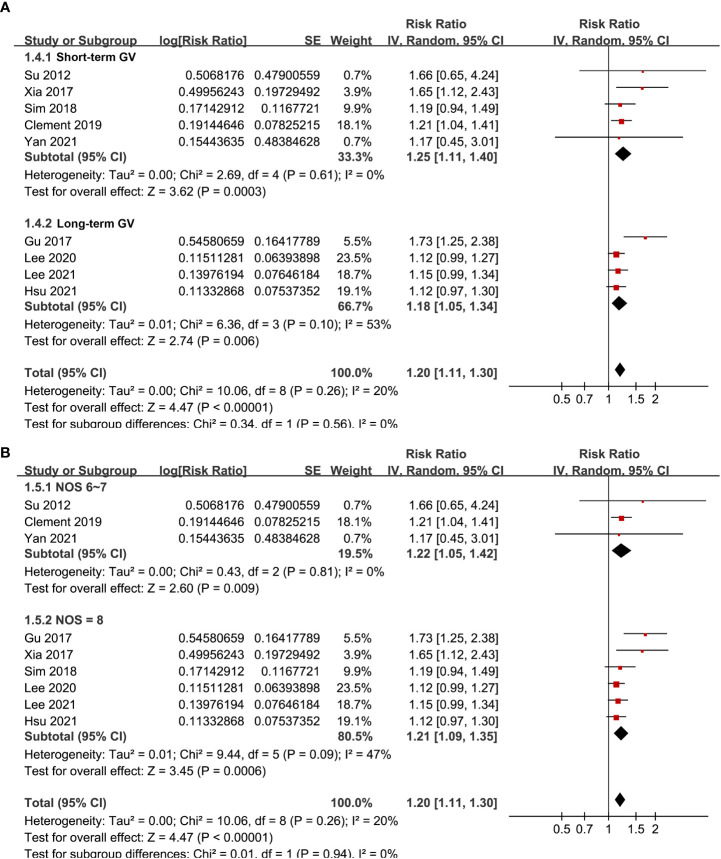
Subgroup analyses for the meta-analysis of the association between GV and the risk of AF. **(A)** subgroup analysis in studies with acute or long-term GV; and **(B)** subgroup analysis in studies with different quality scores.

Pooled results of the nine cohort studies showed that overall, a high GV was associated with a higher risk of AF in adults population (RR: 1.20, 95% CI: 1.11 to 1.30, p < 0.001; **Figure 2**) with mild heterogeneity (p for Cochrane’s’ Q test = 0.26, I^2^ = 20%). Sensitivity analysis by excluding one study at a time did not significantly affect the results (data not shown). Subgroup analyses showed consistent association between a high GV and increased incidence of AF in prospective (RR: 1.29, 95% CI: 1.05 to 1.59, p = 0.01) and retrospective studies (RR: 1.18, 95% CI: 1.08 to 1.29, p = 0.002; p for subgroup difference = 0.44; **Figure 3A**), in diabetic (RR: 1.24, 95% CI: 1.03 to 1.50, p = 0.03) and non-diabetic subjects (RR: 1.13, 95% CI: 1.00 to 1.28, p = 0.05; p for subgroup difference = 0.43; **Figure 3B**), in studies with short-term (RR: 1.25, 95% CI: 1.11 to 1.40, p < 0.001) and long-term GV (RR: 1.18, 95% CI: 1.05 to 1.34, p = 0.006; p for subgroup difference = 0.56; **Figure 4A**), and in studies with quality scores of 6~7 (RR: 1.22, 95% CI: 1.05 to 1.42, p = 0.009) and in those of 8 (RR:1.21, 95% CI: 1.09 to 1.35, p < 0.001; p for subgroup difference = 0.94; **Figure 4B**).

### Publication bias

**Figure 5:**
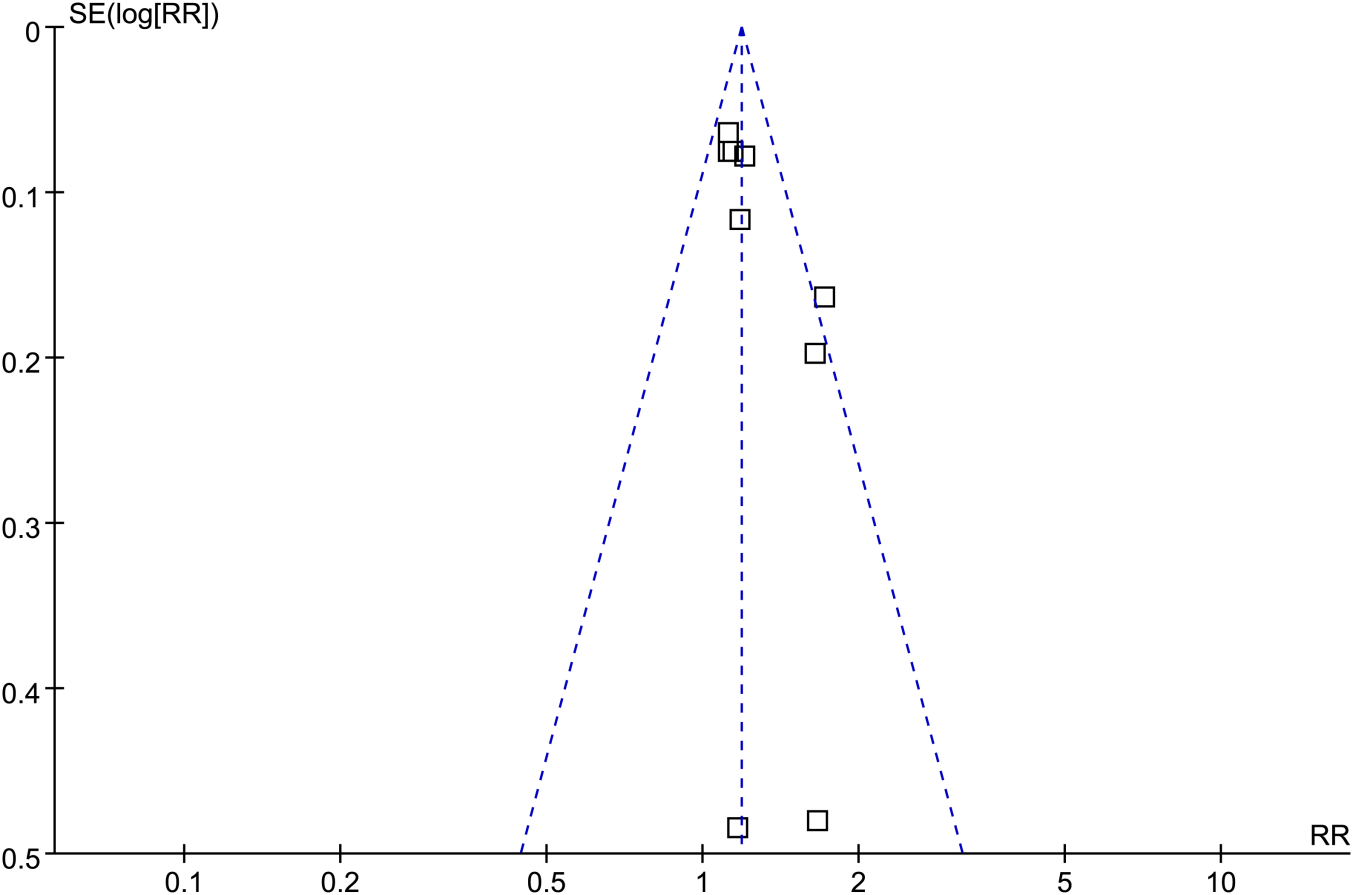
Funnel plots for the meta-analysis of the association between GV and the risk of AF. □ indicates each study included in the meta-analysis.

The funnel plots for the meta-analysis of the association between GV and risk of AF are shown in **Figure 5**. The plots were symmetrical on visual inspection, suggesting low risk of publication bias. Egger’s regression test also suggested a low-risk publication underlying the meta-analysis (p = 0.23).

## Discussion

In this systematic review and meta-analysis, by pooling the results of nine cohort studies, we found that subjects with a high GV at baseline was associated with a higher risk of AF as compared to those with a low GV. The stability of the finding was confirmed by similar results of sensitivity analyses by excluding one study at a time. Moreover, subsequent predefined subgroup analyses showed that the association between a high GV and the increased risk of AF was consistent in prospective and retrospective cohort studies, in diabetic and non-diabetic subjects, in studies with short-term and long-term GV, and in studies with different quality scores. Taken together, these results indicate that a high GV at baseline may be a risk factor of AF in adult population. Although large-scale prospective studies are needed to validate these findings, results of the meta-analysis suggest that measuring GV may be useful to predict the risk of AF.

To the best of our knowledge, this study may be the first systematic review and meta-analysis which investigated the potential relationship between GV and the incidence of AF. We extensively searched for relevant studies in five electronic databases, and nine up-to-date cohort studies were retrieved, with about half of them published within the recent 3 years. Besides, only cohort studies were included, which could therefore provide a longitudinal association between GV and AF. Moreover, multivariate analyses were used to estimate the association between GV and AF among all the included studies, and potential confounding factors such as age, sex, and comorbidities etc. were adjusted, which therefore could indicate an independent relationship between GV and AF. Finally, consistent results were obtained in multiple sensitivity and subgroup analyses, which further confirmed the robustness of the finding. Taken together, this meta-analysis confirmed that a high GV may be a risk factor of AF in adult population, which expanded the previous observations that a high GV is associated with an increased risk of adverse cardiovascular events, such as CAD, stroke, and cardiovascular deaths.

The mechanisms underlying the association between GV and AF remain to be fully understood. An early experimental study in streptozotocin-induced diabetic rats confirmed that glucose fluctuation in uncontrolled diabetes was associated with a high inducibility of AF (35). Meanwhile, an enhanced expression of markers of cardiac fibrosis (collagen type 1, collagen type 3, and α-smooth muscle actin) was also observed in rats with high glucose fluctuation, together with the unregulated markers of reactive oxygen species (ROS) and apoptosis (35). These findings may suggest that glucose fluctuation may accelerate the pathogenesis of AF *via* inducing oxidative stress, cardiomyocyte apoptosis, and atrial fibrosis. In addition, a previous clinical study in patients with newly diagnosed and well-controlled type 2 diabetes showed that a high GV may be associated with reduced cardiac autonomic modulation (36). Interestingly, changes of cardiac autonomic innervation or outflow have been shown to be involved in the pathogenesis of atrial arrhythmias, including AF (37), suggesting GV may induce AF *via* affecting the autonomic function. Finally, emerging evidence from preclinical studies suggested that a high glucose fluctuation may adversely affect the structural and electrical remodeling of the atrium, which may also be the underlying mechanisms for the association between GV and AF (38, 39). Studies are warranted in the future for determination of the molecular signaling pathways involved.

Our study has several limitations. First, the number of the included studies was limited, and some of them were of retrospective design. Therefore, results of the meta-analysis should be validated in large-scale prospective studies. Second, the parameters and definitions of patients with high GV varied among the included studies, which may be a main source of heterogeneity of the meta-analysis. However, as mentioned previously, no consensus has been reached regarding the optimal parameter and corresponding cutoff value for defining subjects with high GV. Additionally, AF is usually classified according to its temporal pattern as paroxysmal, persistent, or permanent (40). Studies are needed to determine if the association between GV and AF is consistent according to the different types of AF. Moreover, subgroup analysis was performed according to the diabetic status of the patients. In prediabetic patients, especially those with impaired glucose tolerance, postprandial glucose fluctuation has been closely related to cardiovascular complications (41). Accordingly, it could be hypothesized that GV in patients with prediabetes may be related to a higher risk of AF. However, to the best of our knowledge, no study has particularly investigated the association between GV and AF in this important population. Studies are warranted to investigate the potential relationship between GV and risk of AF in people with prediabetes. Additionally, although the results regarding the association between GV and AF in this meta-analysis were on the basis of studies with multivariate analyses, there may be residual confounding factors which may affect the association. For example, different antidiabetic strategies may affect the extent of GV differently (42). Accordingly, difference of antidiabetic drugs used in the included patients may affect the association between GV and the risk of AF. Among the strategies with low risk of hypoglycemia, the blood glucose fluctuation of patients is relatively small (43). Most of the new hypoglycemic drugs have low risk of hypoglycemia, and the GV is also small. Insulin-based treatment has a relatively high risk of hypoglycemia and high GV (43). Therefore, the effect of hypoglycemic strategies and drugs on GV and atrial fibrillation is important. On the other hand, insulin-based treatment has a relatively high risk of hypoglycemia and high GV (44). Moreover, the novel hypoglycemic agents such as sodium-glucose cotransporter (SGLT)-2 inhibitors can reduce heart load and improve the prognosis of heart failure, which may also reduce the risk of AF (45). However, for the three studies including diabetic population, none of them investigated the influences of hypoglycemic strategies on the potential relationship between GV and AF. Studies are needed in the future to determine if the potential differences in antidiabetic strategies may modify the relationship between GV and AF. Finally, a causative relationship between a high GV and an increased risk of AF could not be derived because this meta-analysis was based on observational studies. Clinical studies may be considered to determine if reduce the extent of GV could decrease or prevent the incidence of AF.

In conclusion, results of the meta-analysis indicate that a high GV may be a risk factor of AF in adults. Although the results should be validated in prospective studies and the mechanisms should be further determined, results of the meta-analysis suggest that measuring GV may be useful to predict the risk of AF in adult population.

## Data availability statement

The raw data supporting the conclusions of this article will be made available by the authors, without undue reservation.

## Author contributions

WL and GZ designed the study. WL and YW performed database search, literature review, study quality evaluation, data collection, and statistical analyses. WL, YW, and GZ interpreted the results. WL drafted the manuscript. YW and GZ critically revised the manuscript. All authors contributed to the article and approved the submitted version.
